# SARS-CoV-2 Spike Protein Exacerbates Thromboembolic Cerebrovascular Complications in Humanized ACE2 Mouse Model

**DOI:** 10.1007/s12975-024-01301-5

**Published:** 2024-10-02

**Authors:** Stan P. Heath, Veronica C. Hermanns, Maha Coucha, Mohammed Abdelsaid

**Affiliations:** 1 Department of Biomedical Sciences, School of Medicine, Mercer University, Savannah, GA USA; 2 Department of Pharmaceutical Sciences, School of Pharmacy, South University, Savannah, GA USA; 3 Biomedical Sciences Department, Mercer University School of Medicine, 1250 E 66th Street | Savannah, 31404 Macon, GA United States

**Keywords:** SARS-CoV-2 Spike Protein, RAAS Balance, COVID-19-induced Thromboembolic Complications, Vascular-contributes to Cognitive Impairments and Dementia

## Abstract

COVID-19 increases the risk for acute ischemic stroke, yet the molecular mechanisms are unclear and remain unresolved medical challenges. We hypothesize that the SARS-CoV-2 spike protein exacerbates stroke and cerebrovascular complications by increasing coagulation and decreasing fibrinolysis by disrupting the renin-angiotensin-aldosterone system (RAAS). A thromboembolic model was induced in humanized ACE2 knock-in mice after one week of SARS-CoV-2 spike protein injection. hACE2 mice were treated with Losartan, an angiotensin receptor (AT_1_R) blocker, immediately after spike protein injection. Cerebral blood flow and infarct size were compared between groups. Vascular-contributes to cognitive impairments and dementia was assessed using a Novel object recognition test. Tissue factor-III and plasminogen activator inhibitor-1 were measured using immunoblotting to assess coagulation and fibrinolysis. Human brain microvascular endothelial cells (HBMEC) were exposed to hypoxia with/without SARS-CoV-2 spike protein to mimic ischemic conditions and assessed for inflammation, RAAS balance, coagulation, and fibrinolysis. Our results showed that the SARS-CoV-2 spike protein caused an imbalance in the RAAS that increased the inflammatory signal and decreased the RAAS protective arm. SARS-CoV-2 spike protein increased coagulation and decreased fibrinolysis when coincident with ischemic insult, which was accompanied by a decrease in cerebral blood flow, an increase in neuronal death, and a decline in cognitive function. Losartan treatment restored RAAS balance and reduced spike protein-induced effects. SARS-CoV-2 spike protein exacerbates inflammation and hypercoagulation, leading to increased neurovascular damage and cognitive dysfunction. However, the AT_1_R blocker, Losartan, restored the RAAS balance and reduced COVID-19-induced thromboembolic cerebrovascular complications.

## Introduction

The Severe Acute Respiratory Syndrome Corona virus-2 (SARS-CoV-2) is a single-stranded RNA virus responsible for the COVID-19 pandemic. As of 2024, SARS-CoV-2 had killed over a million Americans. COVID-19 causes acute respiratory distress syndrome (ARDS) [1, 2]. ARDS symptoms include severe pneumonia, hypoxemia, and elevated cytokines, known as a cytokine storm. Together, these can result in higher mortality rates, especially in patients with a concomitant cardiovascular disorder such as hypertension, diabetes, or obesity.

Many studies reported that COVID-19 causes neurological symptoms [3, 4]. COVID-19-induced neurological symptoms range from headaches and loss of taste and smell to encephalopathy, cognitive impairments, and stroke [5]. The mechanisms of these neurovascular disorders are unclear and represent a knowledge gap. COVID-19 caused life-threatening coagulopathies such as stroke and deep vein thrombosis [6]. Several reports indicate that these negative thrombotic events are associated with up to one-third of COVID-19 patients [7–9]. The molecular mechanisms behind increased hyper-coagulopathies and impaired fibrinolysis are unclear and require further investigation [10]. The development of vaccination against the SARS-CoV-2 virus has decreased mortality rates. However, long-term COVID-19-induced hyper-coagulopathy and cognitive dysfunction represent a significant medical challenge that requires intensive studies [11].

Angiotensin-converting enzyme-2 (ACE-2) is an enzyme that plays a crucial role in the RAAS balance. ACE-2 degrades angiotensin II, the bioactive form, which can bind to the angiotensin type receptor 1 (AT_1_R) or the angiotensin type receptor 2 (AT_2_R). AT_1_R is more abundant than AT_2_R, and its activation leads to physiological effects such as vasoconstriction, proinflammatory, thrombosis, and generation of reactive oxygen species [12–14]. Activation of the Ang II/AT_1_R axis pathway increases hyper-coagulations via upregulation of the tissue factor (TF), thrombin activation, and platelet aggregation [15, 16]. In contrast, Ang II/AT_2_R axis opposes the effects of AT_1_R activation through vasodilation and anti-inflammatory responses and maintains hemostasis [17–19]. Moreover, ACE-2 degrades Ang II to Ang 1–7, which binds with the MAS receptor. Ang 1–7/MAS receptor axis contributes to neuroprotection through anti-inflammatory and anti-oxidative effects in CNS [20, 21]. ACE-2 binds to the SARS-CoV-2 spike protein and acts as a functional receptor for the spike protein to internalize the viral particle [22]. SARS-CoV-2 downregulates the ACE-2 receptor [4], which decreases Ang II degradation, therefore increasing the RAAS destructive arm with increased activation of the abundant AT_1_R over AT_2_R [23, 24].

This study aims to investigate one of the possible mechanisms by which SARS-CoV-2 Spike protein alters the coagulation and fibrinolysis hemostasis, leading to stroke and neurovascular complications. We hypothesize that the SARS-CoV-2 spike protein exacerbates stroke and neurovascular complications by increasing coagulation and decreasing fibrinolysis via disrupting the RAAS balance.

We examined the effect of SARS-CoV-2 spike protein in a mouse model of humanized ACE-2 knock-in (hACE2 KI) mice. Spike protein binds only with human ACE2 receptors, not murine. hACE2 KI mice expresses humanized ACE2 receptors to replace mice Ace2. This mouse model is among the well-accepted models to study COVID-19 in rodents, used to study antiviral therapies for COVID-19 and SARS-CoV-2 viral replication, transmissibility, and respiratory disorders [25–27]. Our previous studies showed that injection of SARS-CoV-2 spike protein in ACE2 KI mice increases brain inflammation and disrupts RAAS balance in the brain [4, 28]. Spike protein was injected seven days before the induction stroke. Stroke is induced through distal middle cerebral artery (MCA) thromboembolism using ferric chloride (FeCl_3_). We examined cerebral blood flow, infarct-sized and vascular contribution to cognitive impairments, and dementia (VCID) in stroked animals after one week of SARS-CoV-2 spike protein injection.

Our previous studies showed that SARS-CoV-2 spike protein disrupts RAAS balance and increases brain inflammation [4, 28]. Here, our results provide novel evidence that SARS-CoV-2 Spike protein increased coagulation and decreased fibrinolysis in hACE2 KI mice. These effects were accompanied by decreased cerebral blood flow, increased neuronal death, and increased cognitive dysfunctions. Our results showed that using the AT_1_R blocker, Losartan restored the RAAS balance and reduced COVID-19-induced thromboembolic cerebrovascular complications.

## Materials and Methods

### Animals

Transgenic humanized ACE2 KI mice (hACE2 KI), B6.129S2 (Cg)-Ace2^tm1(ACE2)Dwnt^/J mice were purchased from Jackson Laboratory (Jax lab: stock no. 035000, Ellsworth, Maine, USA). hACE2 KI mice have human ACE2 cDNA replacing the endogenous mouse Ace2 sequences. The endogenous Ace2 regulatory elements direct expression of human ACE2, the receptor used for cellular entry by several coronaviruses, including severe acute respiratory syndrome coronavirus-1 (SARS-CoV) and − 2 (SARS-CoV-2) [4, 27]. ARRIVE guidelines 2.0 were followed in conducting animal studies in accordance with the ethical guidelines of the National Institutes of Health Guide for the Care and Use of Laboratory Animals. Mercer University Institutional Animal Care and Use Committee (IACUC) is accredited by the American Association for Accreditation of Laboratory Animal Care, and has approved the animal protocols. A standard mice chow diet was provided to the animals, as was unlimited access to tap water. Twelve-hour cycles of light and dark were used for the mice.

### Animal Treatment

hACE2 mice were randomly assigned to four groups: (1) sham, (2) stroke, (3) stroke + SARSCoV-2 spike protein injection, and (4) stroke + SARSCoV-2 spike protein injection + Losartan. A recombinant protein for SARS-CoV-2 nucleoprotein/spike protein (4ug/animal, Invitrogen, USA, Cat. No. RP-87706) was injected intravenously via jugular vein injection seven days prior to MCA/FeCl_3_ thromboembolic surgery. Sham animals were exposed to the MCA/FeCl_3_ thromboembolic surgery without FeCl_3_ treatment.

Losartan (10 mg/kg body weight, Tokyo Chemical Industry, Tokyo, Japan, Cat. No. L0232) was added to the water supply of the animal cages. Losartan was first administered immediately after the injection of the recombinant spike protein for SARS-CoV-2. Losartan is affordable compared to other ARBs, making it accessible for long-term use. It is water-soluble, which facilitates its administration and absorption. Also, its solution remains stable for several days, ensuring consistent efficacy. Losartan has relatively higher lipophilicity than other ARBs, increasing its ability to cross the blood-brain barrier. Finally, Losartan is a promising therapeutic agent for managing neurological disorders as it has clinically proven neuroprotective effects in several neurodegenerative disorders [29, 30].

### MCA/FeCl_3_ Induced Thromboembolic Model

Eight-week-old male hACE2 KI mice weighing 20–25 g were randomly assigned for surgery. Mice were anesthetized using a mixture of Ketamine and Xylazine intraperitoneal (IP) injection (100 mg/kg ketamine and 10 mg/kg Xylazine). Long-acting Buprenorphine (0.1 mg/kg) IP injection was also given before surgery to decrease postoperative pain. A vertical incision was made between the eye and ear to locate the temporal muscle. A horizontal incision is then made on the lateral ridge of the frontal bone, and a vertical incision from the rostral-most point of the parietal bone is made to expose the skull. The distal trunk of the middle cerebral artery is then located and exposed by drilling through the mouse skull. The meninges were removed to expose the artery, and the transient focal ischemia model was initiated by using fresh FeCl_3_ (20%) soaked filter paper for five minutes. After removal, sterile warm saline was applied to the area for 10 min and then dried with sterile gauze. The temporal muscle was then placed back, the mouse was sutured, and the surgical area was disinfected with a povidone-iodine solution. The mouse was then monitored under a heat lamp until recovery.

### Assessment of Cerebral Blood Flow

Cerebral blood flow was measured with the RFLSI III Laser Speckle Imaging System (RWD, San Diego, CA, USA). Mice were anesthetized using isoflurane, and a vertical incision was made from the sagittal suture to the interferential suture on the mouse’s skull. RWD laser speckle imaging was used to record the cerebral blood flow. LSCI software (Version 5.0) was used to measure the perfusion per area at hours 1, 2, 3, 6, and 24. Perfusion was represented as a percentage ratio of the stroke to the non-stroke hemisphere of the brain.

### Assessment of Infarct Volume

hACE2 mice brains were collected 24 h after MCA/FeCl_3_ thromboembolic model surgery. These brains were sectioned into five coronal 2 mm thick slices using an acrylic brain matrix. Coronal brain slices were examined by staining with 2% 2,3,5-triphenyl tetrazolium chloride (TTC) and incubating for 15 min in a humidified incubator (37%C, 5% CO2). Sections were photographed with units of measure to be quantified blindly using Image-J software.

### Assessment of Cognitive Functioning

Memory and learning functions were assessed at baseline, before SARS-CoV-2 spike protein injection, and 24 h after MCA/FeCl_3_ thromboembolic surgery using Novel Object Recognition testing (NOR) [31, 32].

The NOR test is a 3-stage test used to examine recognition memory. The first stage involves habituation, with the mouse being exposed to its testing environment. The second is the familiarization stage, which involves the mouse being exposed to two identical objects. The final phase is the testing phase, which involves the replacement of one of the familiar objects with a novel object. The testing phase is recorded using ANY-maze software. Time spent around the novel object, the ratio of time spent investigating the novel object to the familiar object, and the total distance traveled were measured using the ANY-maze software recording and analyzed blindly.

### Cell Culture

Primary human brain microvascular endothelial cells (HBMECs, from Angio-Proteomie, Boston, MA, USA, Cat. No. cAP-0002) were grown to confluency in complete media (MCDB-131 Complete, VEC Technologies, Inc., Rensselaer, NY, USA). HBMECs were switched to serum-reduced media and treated with SARS-CoV-2 recombinant spike protein (100 µM) with or without losartan (100 µM) for 24 h. The Next day, HBMECs were either kept under normoxia conditions (room air 21% oxygen) or placed in a hypoxia chamber (0.1% oxygen, BioSpherix, ProOx Model 110) for 6 h. Cells were collected immediately once removed from the hypoxia chamber.

### Polymerase Chain Reaction

RT-PCR was performed as described previously [4]. RNA was isolated using Triazole (Thermo-Fisher, USA, Cat. No. AC345480250) and quantified using a Thermo Scientific NanoDrop 2000 C Spectrophotometer (Thermo Scientific, USA). cDNA was prepared from isolated RNA using SuperScript IV VILO Master mix with an ezDNase kit (Invitrogen, USA, Cat. No. 11766050). The Quant Studio™ 3 Real-Time PCR System (Applied Biosystems, Thermo Scientific, USA) was utilized to run qRT-PCR using PowerUp SyBR Green Master mix (Applied Biosystems, Thermo Scientific, USA, Cat No. A25742). All primer sequences used are detailed in (Table 1). GAPDH was consistently used as the reference gene for normalization.

**Table 1:** PCR primers

Primer	Species	FWD sequence	REV sequence
ACE2	Human	5’-TCC ATT GGT CTT CTG TCA CCC G-3’	5’-AGA CCA TCC ACC TCC ACT TCT C-3’
	Mouse	5’-TCC ATT GGT CTT CTG CCA TCC G-3’	5’-AGA CCA TCC ACC TCC ACT TCT C-3’
AT_1_R	Human	5’-CAG CGT CAG TTT CAA CTT GTA CG-3’	5’-GCA GGT GAC TTT GGC TAC AAG C-3’
	Mouse	5’-GCC ATT GTC CAC CCG ATG AAG T-3’	5’-ACA CAT TTC GGT GGA TGA CGG C-3’
AT_2_R	Human	5’-CCA TGT TCT GAC CTT CCT GGA TG-3’	5’-CGG ATT AAC GCA GCT GTT GGT G-3’
	Mouse	5’-CGT GAC CAA GTC CTG AAG ATG G-3’	5’-GGA AGT GCC AGG TCA ATG ATG ACT G-3’
GAPDH	Human	5’-GTC TCC TCT GAC TTC AAC AGC G-3’	5’-ACC ACC CTG TTG CTG TAG CCA A-3’
	Mouse	5’-CAT CAC TGC CAC CCA GAA GAC TG-3’	5’-ATG CCA GTG AGC TTC CCG TTC AG-3’
Il-1β	Human	5’-CCA CAG ACC TTC CAG GAG AAT G-3’	5’-GTG CAG TTC AGT GAT CGT ACA GG-3’
	Mouse	5’-TGG ACC TTC CAG GAT GAG GAC A-3’	5’-GTT CAT CTC GGA GCC TGT AGT G-3’
I-6	Human	5’-AGA CAG CCA CTC ACC CTCT TAC G-3’	5’-TTC TGC CAG TGC CTC TTT GCT G-3’
	Mouse	5’-TAC CAC TTC ACA AGT CGG AGG C-3’	5’-CTG CAA GTG CAT CAT CGT TGT TC-3’
MASR	Human	5’-CAG CAC CAT CTT GGT CGT GAA G-3’	5’-CAG CAG GTA AAG GAG TCT CAT GG-3’
	Mouse	5’-CTG ACA GCC ATC AGT GTG GAG A-3’	5’-GTG GTC ACC AAG CAC GAA AGT G-3’
NFκB	Mouse	5’-GCT GCC AAA GAA GGA CAC GAC A-3’	5’-GGC AGG CTA TTG CTC ATC ACA G-3’
TNFα	Human	5’-CTC TTC TGC CTG CTG CAC TTT G-3’	5’-ATG GGC TAC AGG CTT GTC ACT C-3’
	Mouse	5’-GGT GCC TAT GTC TCA GCC TCT T-3’	5’-GCC ATA GAA CTG ATG AGA GGG AG-3’

### Immunoblotting

RIPA buffer (Millipore, Billerica, MA, USA, Cat# 3P 20188) was used to extract protein from the hACE2 brain and HBMECs. Equal protein loads were separated on 10% SDS-polyacrylamide gel utilizing the Mini PROTEAN Tetra Cell SDS-PAGE Gel electrophoresis kit (Biorad Laboratories Inc, Hercules, CA). Gels were transferred onto nitrocellulose membranes using the Bio-Rad Trans-Blot Turbo (Biorad Laboratories Inc, Hercules, CA, USA). Membranes were blocked with 5% milk and incubated overnight with primary antibodies. Membranes were washed and incubated with an appropriate horseradish peroxidase-conjugated secondary antibody. Membranes were reacted with Western chemiluminescent HRP Substrate (Millipore, USA) and imaged using the iBright Imaging system (Invitrogen Thermo Fisher Scientific, USA, Model FL1500). Image-J software (Version 1.54 g) was used to measure band intensity. β-actin was used for normalization. All antibodies used are listed in (Table 2).

**Table 2:** Antibodies

Primary Antibody	Company	CAT/ Lot/ Prod	Recommended Concentration	Dilution Immunoblot	Secondary Antibody
AT_1_R	Invitrogen	PA5-20812	1 mg/mL	1:1000	Rabbit
AT_2_R	Novus	NBP1-60097	1 mg/mL	1:1000	Rabbit
Β-actin	R&D	MAB8929	1 mg/mL	1:1000	Mouse
PAI-1	Biotechne	MAB-17861-100	1 mg/mL	1:1000	Rabbit
TNFα	NOVUS	NBP1-19532	1 mg/mL	1:500	Rabbit
TF-III	R&D	MAB-3178	1 mg/mL	1:1000	Mouse

### Statistical Analysis

GraphPad prism version 10 or higher was employed for all data analysis. For animal studies, the sample size was determined from our previous work [4]. One-way ANOVA was utilized to assess mean differences between (1) sham, (2) stroke, (3) stroke + SARS-CoV-2 spike protein injection, and (4) stroke + SARS-CoV-2 spike protein injection + Losartan. Significance was determined at *P* < 0.05. Data is presented as mean ± standard deviation. A Tukey’s post-hoc test was used to adjust for the multiple comparisons to assess significant interaction effects between groups.

## Results

### SARS-CoV-2 Spike Protein Intensifies RAAS Imbalance after Ischemic Insult

**Fig. 1:**
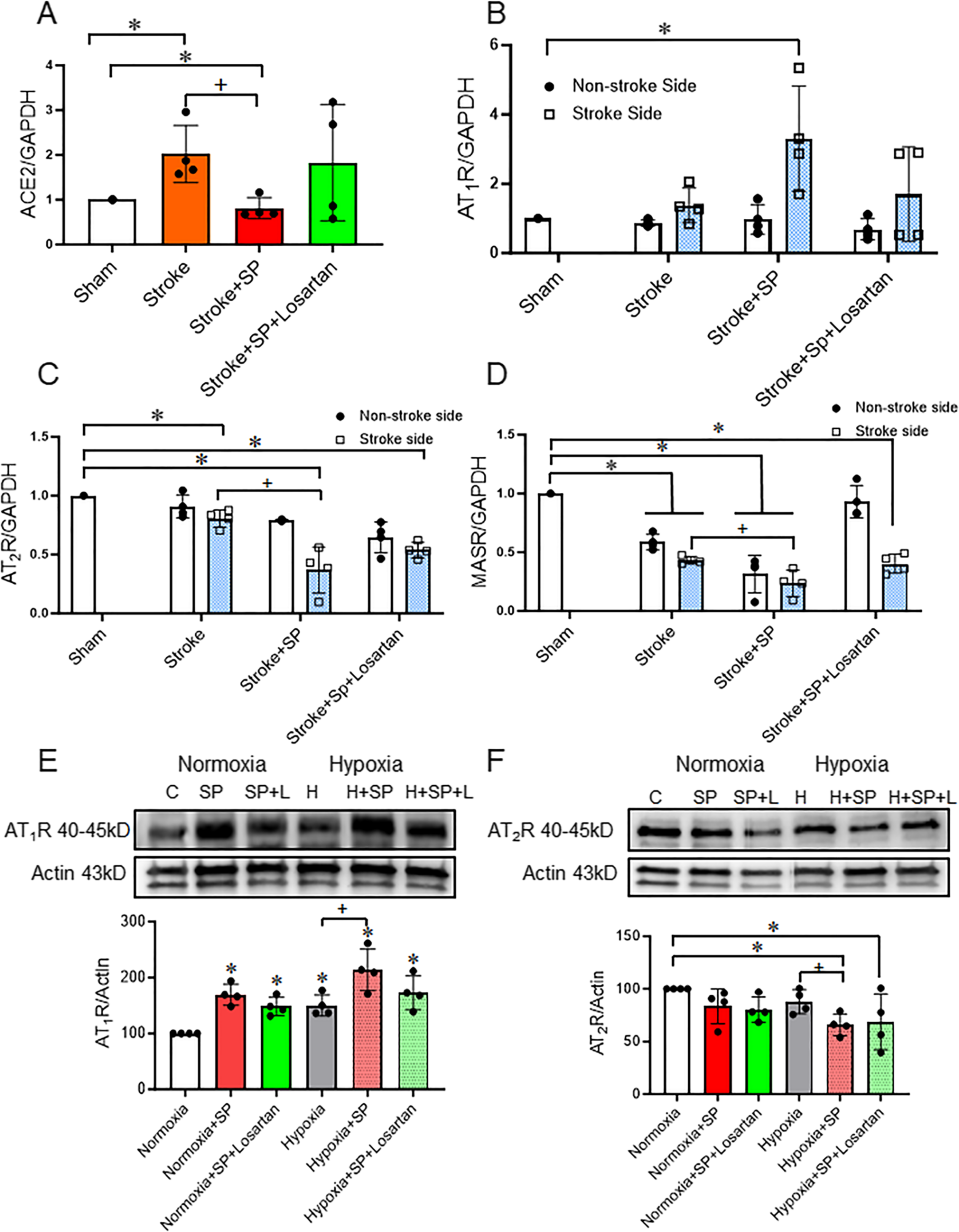
SARS-CoV-2 spike protein intensifies RAAS imbalance after ischemic insult. We assessed the effect of SARS-CoV-2 spike protein on RAAS balance in ACE2 KI mice brain and HBMECs after ischemic insult. ACE2 KI mice were injected with SARS-CoV-2 spike protein (SP) one week before thromboembolic model induction. **(A)** RT-PCR analysis for ACE2 gene expression in brain homogenate. **(B)** RT-PCR analysis for AT_1_R gene expression in brain homogenate. **(C)** RT-PCR analysis for AT_2_R gene expression in brain homogenate. **(D)** RT-PCR analysis for MAS receptor gene expression in brain homogenate. (A-D, One-way ANOVA, *p < 0.05 vs. sham, ^+^*P* < 0.05 vs. stroke (Stroke side), *n* = 4). HBMECs were treated with SARS-CoV-2 spike protein for 24 h before exposure to hypoxia. **(E)** Western blot representative and analysis for AT_1_R expression in HBMECs. **(F)** Western blot representative and analysis for AT_2_R expression in HBMECs. (E-F, One-way ANOVA, * *p* < 0.05 vs. control, ^+^*P* < 0.05 vs. hypoxia, *n* = 4). Our results showed that pretreatment with SARS-CoV-2 spike protein intensifies RAAS imbalance after ischemic insult. (C: Control, H: Hypoxia, SP: Spike protein, SP + L: Spike protein + Losartan)

We assessed the effect of SARS-CoV-2 spike protein on the gene expression of both RAAS arms in hACE2 KI mice brain and human microvascular endothelial cells (HBMECs) subjected to ischemic insult. We analyzed the gene expression of ACE2 in brain homogenate. Moreover, we measured AT_1_R, AT_2_R, and MASR in both the contralateral and ipsilateral brain homogenate using qRT-PCR. Singh et al. showed that a transient MCA occlusion stroke model increases ACE2 expression in the lung of mice [33]. Here, we showed that the MCA/FeCl_3_ transient stroke model increases the ACE2 expression in the brain of hACE2 KI mice (Fig. 1A). We previously showed that SARS-CoV-2 spike protein downregulated ACE2 expression in hACE2 KI mice brains [4, 28]. In the current study, a similar finding was reported. SARS-CoV-2 spike protein downregulates ACE2 gene expression in hACE2 KI brains after the thromboembolic occlusion model (Fig. 1A). Losartan treatment restored ACE2 gene expression after SARS-CoV-2 spike protein injection with the thromboembolic occlusion model. Next, we evaluated the AT_1_R, AT_2_R, and MASR in both stroke and non-stroke hemispheres. Our results showed that ischemic insult increases the expression of AT_1_R by 2-fold. Pre-injection of SARS-CoV-2 spike protein before the ischemic insult further increases the expression of AT_1_R to 4-fold in the brain of hACE2 KI mice (Fig. 1B). In contrast, SARS-CoV-2 spike protein decreased the gene expression of the RAAS protective arms AT_2_R and MASR (Fig. 1C-D). These results were confirmed in HBMECs that were treated with SARS-CoV-2 spike protein and subjected to hypoxia to mimic the ischemic insult. Our results showed that SARS-CoV-2 spike protein increases AT_1_R expression and decreases AT_2_R expression under normoxic conditions. The addition of ischemic insult further augments SARS-CoV-2 spike protein-induced effects. Losartan treatment reduced spike protein-induced effects but did not reach significance. (Fig. 1E-F).

### Increased Inflammation After a Double Hit, SARS-CoV-2 Spike Protein Followed By Ischemic Insult Exacerbates Inflammation in HACE2 Brains

**Fig. 2:**
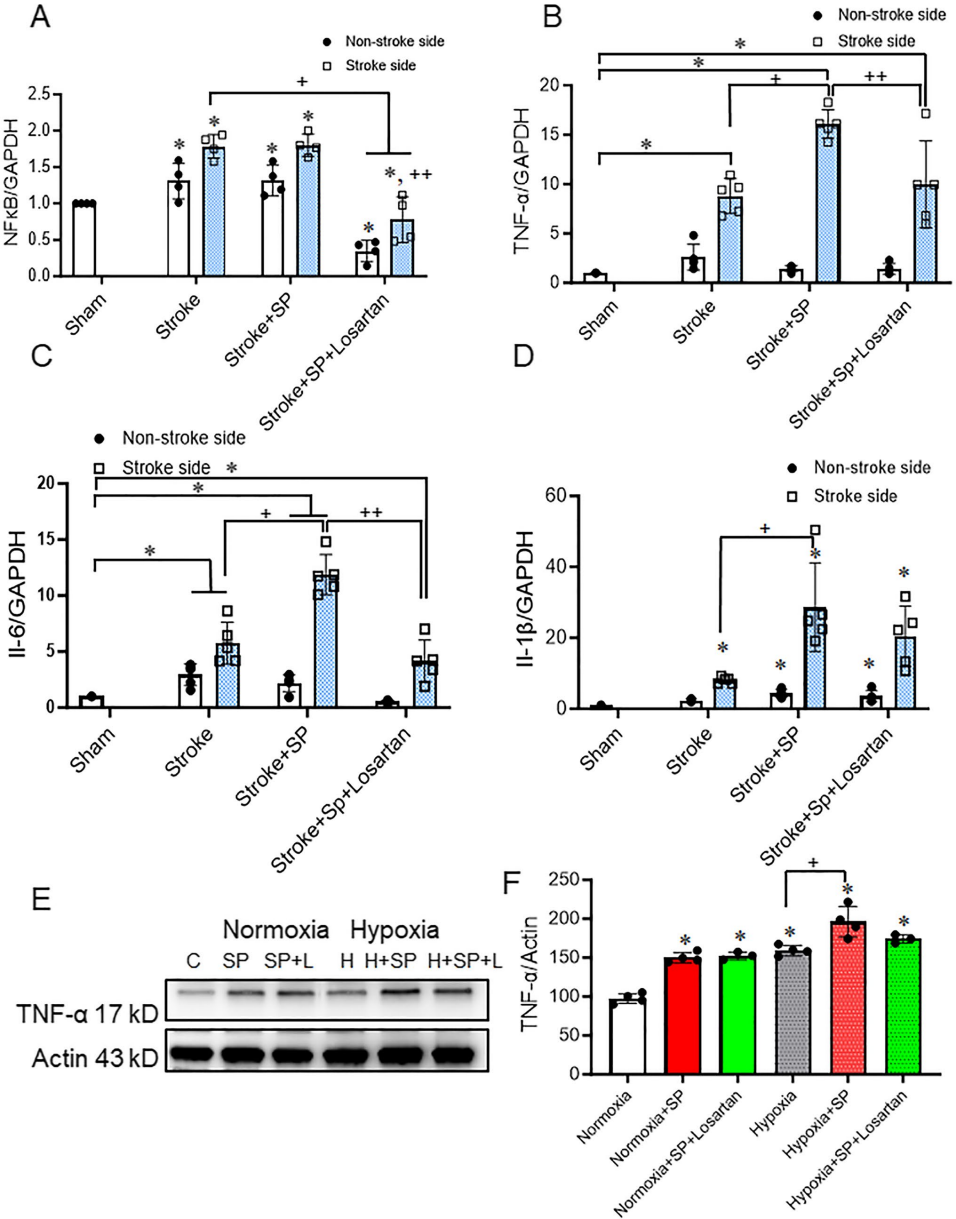
SARS-CoV-2 spike protein exacerbates inflammation in hACE2 brains. We assessed the effect of pre-injecting SARS-CoV-2 spike protein on brain inflammation before applying ischemic insult. ACE2 KI mice were injected with SARS-CoV-2 spike protein (SP) one week before thromboembolic model induction. **(A)** RT-PCR analysis for NF_Ϗ_B gene expression in brain homogenate. **(B)** RT-PCR analysis for TNF-α gene expression in brain homogenate. **(C)** RT-PCR analysis for Il-6 gene expression in brain homogenate. **(D)** RT-PCR analysis for Il-1β gene expression in brain homogenate. (A-D, One-way ANOVA, *p < 0.05 vs. sham, ^+^*P* < 0.05 vs. stroke (Stroke side), ^++^*P* < 0.05 vs. stroke + spike protein (Stroke side), *n* = 4–5). HBMECs were treated with SARS-CoV-2 for 24 h before exposure to hypoxia. **(E)** Western blot representative for TNF-α expression. **(F)** Western blot representative analysis for TNF-α expression in HBMECs. (One-way ANOVA, * *p* < 0.05 vs. normoxia, ^+^*P* < 0.05 vs. hypoxia, *n* = 3–4). Our results showed that SARS-CoV-2 intensifies inflammation when coincident with ischemic insult, and losartan treatment significantly reduced its effects. (C: Control, H: Hypoxia, SP: spike protein, SP + L spike protein + Losartan)

Stroke is known to increase brain inflammation [34]. We have previously shown that intravenous injection of SARS-CoV-2 spike protein increases inflammation in the brain of hACE2 KI mice [4]. Here, we examined the effect of SARS-CoV-2 spike protein injection coincident with ischemic insult on brain inflammation. Our results showed that SARS-CoV-2 spike protein pre-injection significantly intensifies inflammation after induction of thromboembolic occlusion model. Ischemic insults increased inflammation in both ipsilateral and contralateral brain hemispheres. Pre-injection of SARS-CoV-2 spike protein significantly increased NF_Ϗ_B, TNF-α, Il-1β, and Il-6 gene expression in the brains of hACE2 KI mice. Losartan showed a modest but significant effect in reducing the SARS-CoV-2 spike protein-induced effect (Fig. 2A-D). Our results showed similar findings when we examined the impact of SARS-CoV-2 spike protein on HBMCE subjected to hypoxia. SARS-CoV-2 spike protein significantly increased the expression of TNF-α under normoxia conditions. Pretreatment of HBMECs with SARS-CoV-2 spike protein prior to hypoxia further significantly increased endothelial cell inflammation (Fig. 2E, F).

#### SARS-CoV-2 Spike Protein Disrupts Coagulation Homeostasis

**Fig. 3:**
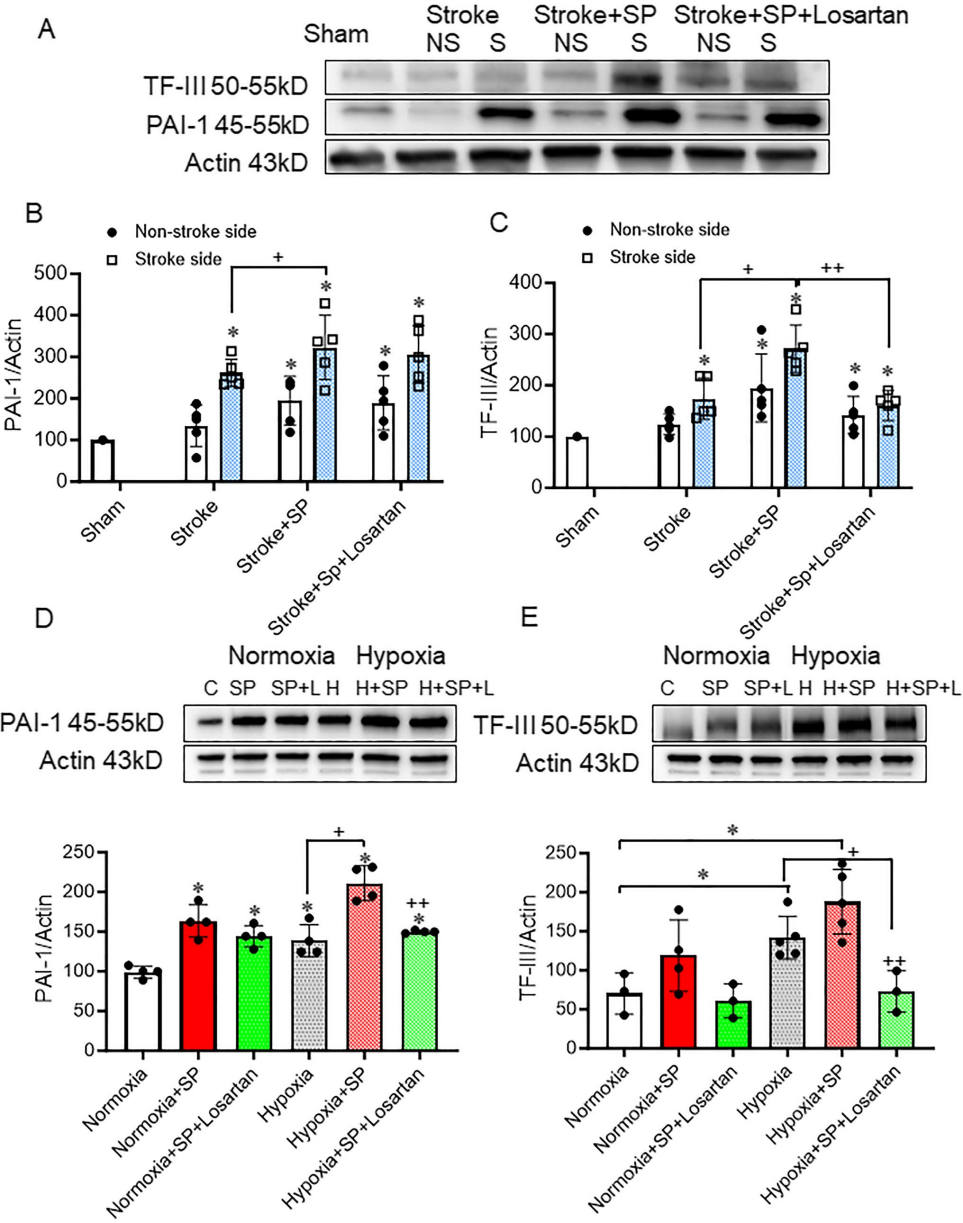
SARS-CoV-2 spike protein disrupts coagulation homeostasis. We assessed the effect of pre-injecting SARS-CoV-2 spike protein (SP) on coagulation homeostasis prior to applying ischemic insult. ACE2 KI mice were injected with SARS-CoV-2 spike protein (SP) one week before thromboembolic model induction. **(A)** Western blot representative for TF-III and PAI-1. **(B)** Western Blot analysis for PAI-1 expression in brain homogenate. **(C)** Western Blot analysis for TF-III expression in brain homogenate. (B-C, One-way ANOVA, *p < 0.05 vs. sham, ^+^*P* < 0.05 vs. stroke (Stroke side), ^++^*P* < 0.05 vs. stroke + spike protein (Stroke side), *n* = 4–5). HBMECs were treated with SARS-CoV-2 spike protein for 24 h before exposure to hypoxia. **(D)** Western blot representative and analysis for PAI-1. **(E)** Western blot representative and analysis for TF-III (One-way ANOVA, * *p* < 0.05 vs. control, ^+^*P* < 0.05 vs. hypoxia, ^++^*P* < 0.05 vs. hypoxia + spike protein, *n* = 3–4). Our results showed that SARS-CoV-2 increases coagulation and decreases fibrinolysis. Losartan treatment significantly decreased spike protein-induced hypercoagulation. (C: Control, H: Hypoxia, SP: spike protein, SP + L spike protein + Losartan)

We examined the effect of SARS-CoV-2 spike protein on the prothrombotic state. The disruption of the endothelial matrix leads to the release of tissue factor III (TF−III), which activates the coagulation cascade, resulting in thrombin activation. Our results showed that the thromboembolic stroke model exacerbated TF−III protein expression in hACE2 KI mice brain homogenate. The injection of spike protein before stroke further escalated TF−III expression. (Fig. 3A, C). PAI-1 inhibits the conversion of plasminogen to plasmin by urokinase-plasminogen activator and tissue-type plasminogen activator (tPA), thereby hindering fibrin degradation. Our results showed that the thromboembolic stroke model increased PAI-1, and the expression of PAI-1 was further increased by the injection of the spike protein seven days before the stroke. (Fig. 3A, B). Similar findings were observed in the HBMECs when exposed to hypoxic conditions with and without SARS-CoV-2 spike protein. Spike protein favors clot formation by increasing coagulation and decreasing fibrinolysis. Losartan treatment significantly decreased spike protein-induced hypercoagulation. (Fig. 3D, E).

### SARS-CoV-2 Spike Protein Decreases Cerebral Blood Flow Following Stroke

**Fig. 4:**
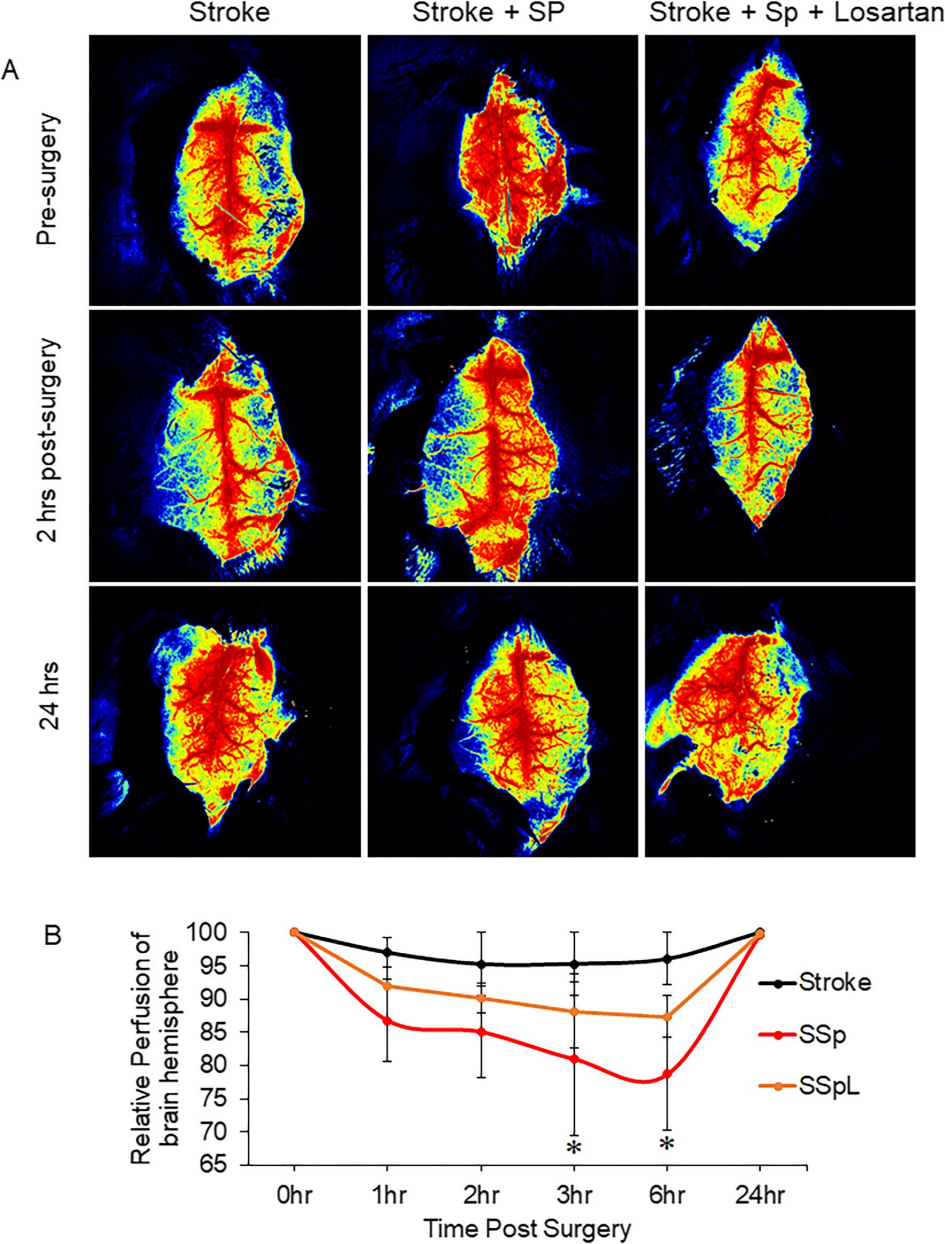
SARS-CoV-2 spike protein decreases cerebral blood flow following stroke. We assessed the effect of pre-injecting SARS-CoV-2 spike protein (SP) on cerebral blood flow and vascular recanalization following a thromboembolic model. ACE2 KI mice were injected with SARS-CoV-2 spike protein (SP) one week before thromboembolic model induction. Laser speckle imaging was employed to measure cerebral blood flow at various intervals of 1, 2, 3, 6, and 24 h post-surgery. **(A)** Cerebral blood flow representative images. **(B)** Cerebral Blood flow and recanalization analysis. (One-way ANOVA, *p < 0.05 vs. stroke, *n* = 4–6). Our results showed that pre-treatment with SARS-COV-2 spike protein significantly decreased cerebral blood flow and increased vascular recanalization time. Treatment with Losartan reduced spike protein effects. (Stroke, SSP : Stroke + Spike protein, SSPL: Stroke + Spike protein + Losartan)

The thromboembolic occlusion was induced in hACE2 KI mice using the MCA/FeCl_3_ model. SARS-CoV-2 spike protein was injected intravenously one week before stroke induction. Laser speckle imaging was employed to measure cerebral blood flow at 1, 2, 3, 6, and 24 h post-surgery. Our results showed that the thromboembolic model decreased cerebral blood flow in the stroke hemisphere compared to the contralateral hemisphere. The spontaneous recanalization occurred, and cerebral blood flow was restored within six hours of the embolic model. The pre-treatment with SARS-COV-2 spike protein showed a significant decrease in cerebral blood flow and increased vascular recanalization time to over 6 h. Treatment with Losartan helped in faster restoration of cerebral blood flow and decreased recanalization time compared to spike protein (Fig. 4A, B).

### SARS-CoV-2 Spike Protein Increases Infarction Volume Following Stroke

**Fig. 5:**
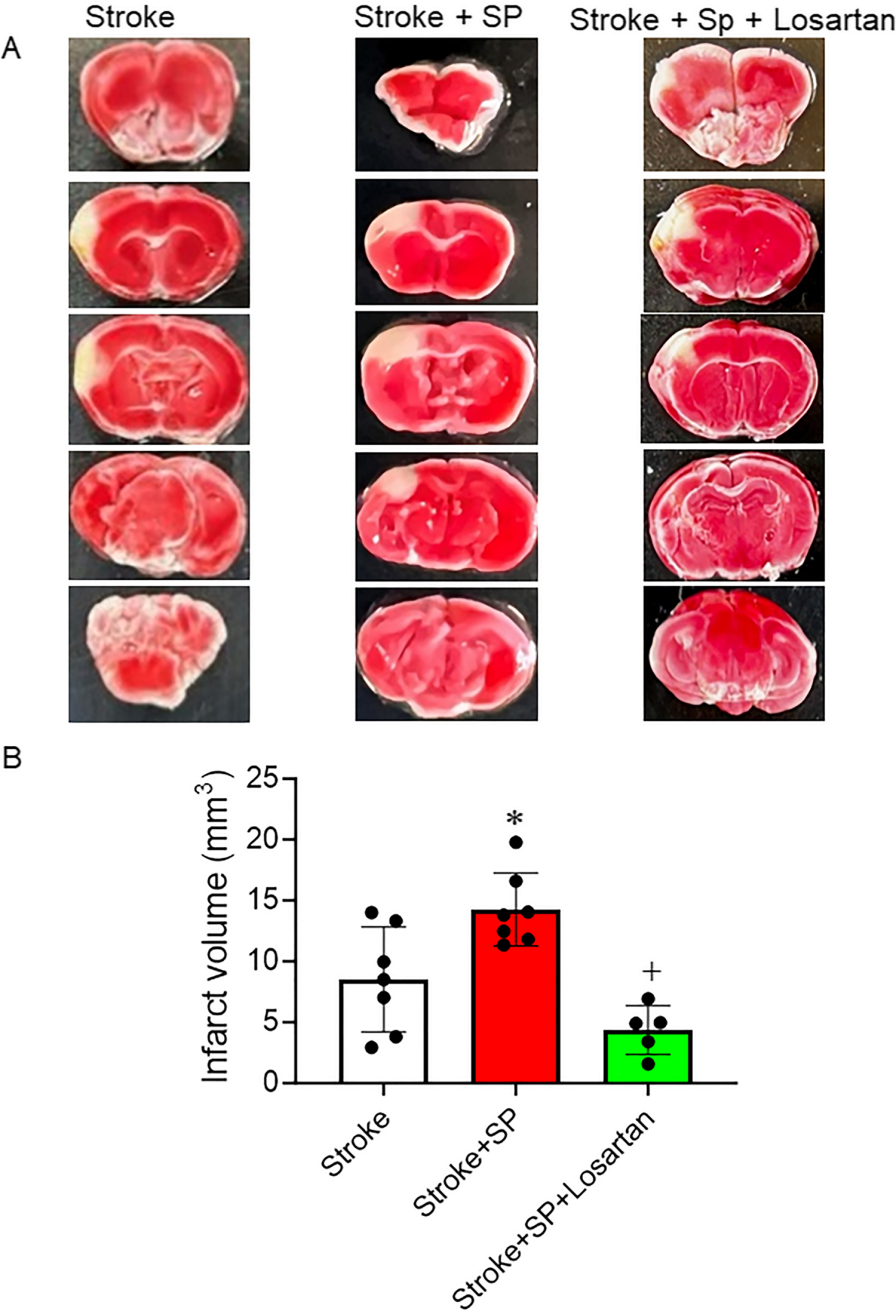
SARS-CoV-2 spike protein decreases infarct volume following stroke. We assessed the effect of pre-injecting SARS-CoV-2 spike protein (SP) on infarct volume following a thromboembolic stroke model. ACE2 KI mice were injected with SARS-CoV-2 spike protein (SP) one week before thromboembolic model induction. Brains were isolated and stained with TTC stain to detect infarct volume. **(A)** Representative images. **(B)** Infarct volume analysis. (A-B, One-way ANOVA, *p < 0.05 vs. stroke, ^+^*P* < 0.05 vs. stroke + spike protein, *n* = 5–8). Our results showed that pre-treatment with SARS-CoV-2 spike protein significantly increased infarct volume compared to stroke. Treatment with Losartan significantly reduced infarct volume and reduced spike protein effects. (Stroke + SP : Stroke + Spike protein, Stroke + SP + Losartan : Stroke + Spike protein + Losartan)

ACE2 KI mice were injected with SARS-CoV-2 spike protein one week before thromboembolic model induction. Brains were isolated and stained with TTC stain to detect dead infarct volume. Our results showed that pre-treatment with SARS-CoV-2 spike protein significantly increased infarct volume compared to stroke. Treatment with the AT_1_R blocker, Losartan, significantly reduced infarct volume and reduced spike protein effects (Fig. 5A, B).

### SARS-CoV-2 Spike Protein Aggravates Cognitive Dysfunction After Thromboembolic Occlusion Model

**Fig. 6:**
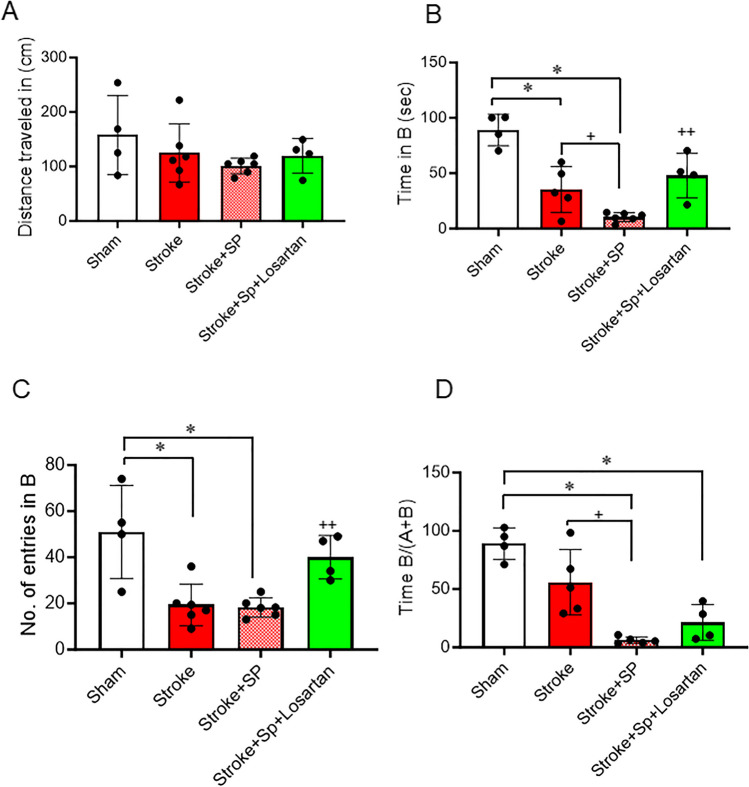
SARS-CoV-2 spike protein aggravates cognitive dysfunction after thromboembolic occlusion model. We assessed the effect of pre-injecting SARS-CoV-2 spike protein (SP) on cognitive dysfunction following a thromboembolic stroke model. ACE2 KI mice were injected with SARS-CoV-2 spike protein (SP) one week before thromboembolic model induction. A novel object recognition test was used to assess cognitive dysfunction. **(A)** Total distance traveled. **(B)** Time spent investigating the new object. **(C)** Number of entries to novel object zone. **(D)** Time spent on novel object to total time spent in object recognition. (A-D, One-way ANOVA, *p < 0.05 vs. sham, ^+^*P* < 0.05 vs. stroke, ^++^*P* < 0.05 vs. stroke + spike protein, *n* = 4–6). Our results showed that pre-treatment with SARS-CoV-2 spike protein significantly increased cognitive dysfunction compared to stroke. Treatment with Losartan significantly reduced spike protein-induced cognitive impairments. (SP: Spike protein)

We have previously shown that SARS-CoV-2 causes cognitive dysfunction in hACE2 KI mice [4]. Here, we assessed the effect of SARS-CoV-2 spike protein on cognitive function after induction of the thromboembolic model. hACE2 KI mice were injected with SARS-CoV-2 spike protein one week before thromboembolic model induction. The Novel Object Recognition test was used to assess memory and learning in hACE2 KI mice. hACE2 KI mice were familiarized with two identical objects. On test day, one of the objects was replaced with a novel object. Time spent investigating the novel object indicates memory and learning cognition in the mouse. We assessed the total distance traveled for each animal to exclude motor dysfunction. Our results showed no significant changes between groups in the total distance traveled (Fig. 6A). There was a significant decrease in both the number of entries into the novel object zone and the time spent interacting with the novel object in the mice subjected to stroke. A similar decrease in the number of entries was observed in the group that received a prior spike protein injection before stroke. However, the spike protein pre-injection further exacerbated the reduction in time spent with the novel object. Treatment with Losartan significantly improved hACE2 KI mice’s cognitive function, as evidenced by the increase in both the time spent with the novel object and the number of entries into the novel object zone (Fig. 6B-D).

## Discussion

The COVID-19 pandemic caused over one million American deaths. Survivors suffered a wide range of cerebrovascular complications, including stroke and cognitive impairments. The potential mechanisms underlying these disorders are not fully understood. Our study tests one of the possible mechanisms by which SARS-CoV-2 disrupts coagulation hemostasis and increases cerebrovascular thromboembolic complications. The main finding of our study is that the SARS-CoV-2 spike protein disrupts the renin-angiotensin-aldosterone system (RAAS) balance in the brain vasculature. The SARS-CoV-2 spike protein increases Ang II/AT_1_R signaling in the brain’s endothelial cells at the expense of the Ang II/AT_2_R protective arm, which increases brain inflammation. Moreover, our results showed that RAAS imbalance contributes to increased coagulation and decreased fibrinolysis, exacerbating stroke, and vascular contribution to cognitive impairments and dementia (VCID). Lastly, restoration of RAAS balance using AT_1_R blocker, Losartan, decreased SARS-CoV-2 spike protein-induced thromboembolic cerebrovascular complications.

With the development of effective COVID-19 vaccines and the reduction of COVID-19 mortality, many COVID-19-induced neurovascular complications are more clinically visible. COVID-19 causes a wide range of neurological disorders [35–38]. These neurological disorders ranged from headaches, loss of smell, and altered mental status to encephalitis and ischemic stroke. COVID-19 not only increased the ischemic stroke rates in the general population but also increased mortality and severity in stroke patients. COVID-19 infections worsen stroke outcomes, especially in patients with a prevalence of vascular risk factors, including age, male gender, hypertension, hyperlipidemia, ischemic heart disease, and diabetes mellitus [4, 11]. There are multiple hypotheses that account for COVID-19’s increased thromboembolic events in patients, including increased vascular inflammation and cytokine storms, endothelial dysfunction, pericyte loss, blood-brain barrier dysfunction, and neuroinflammation [39–43]. Here, we hypothesized that SARS-CoV-2 spike protein exacerbates stroke and cerebrovascular complications by increasing coagulation and decreasing fibrinolysis via disrupting the RAAS balance.

SARS-CoV-2 spike protein binds with the ACE-2 receptor as one of the binding sites to achieve cell entry. ACE-2 plays a crucial role in the degradation of Ang II, the bioactive form of the RAAS, to Ang 1–7. We have previously shown that SARS-CoV-2 spike protein decreases ACE-2 expression and increases Ang II/AT_1_R downstream inflammatory signaling and endothelial cell apoptosis in the brain of humanized ACE2 knock-in mice [4]. Our study also showed that spike protein significantly downregulated the RAAS protective arm with decreased AT_2_R and MAS receptor expression ^3^. Singh et al. showed that transient MCA occlusion increases ACE-2 expression in mice, which might increase the binding affinity to SARS-CoV-2 spike protein [33]. In the present study, we provide novel evidence that the SARS-CoV-2 spike protein-induced RAAS imbalance increases coagulation and decreases fibrinolysis, which worsens ischemic stroke outcomes in a distal middle cerebral artery (MCA) thromboembolic model.

Our results showed that SARS-CoV-2 spike protein increases coagulation via increased Tissue Factor III (TF-III) expression in brain endothelial cells. TF-III is expressed and activated by endothelial cells, vascular smooth muscle cells, and monocytes [44]. TF-III activates the extrinsic coagulation pathway that activates factor VII, which in turn catalyzes the conversion of the inactive factor X into the active factor Xa. In addition, SARS-CoV-2 spike protein increased the expression of Plasminogen activator inhibitor-1 (PAI-1), a serine protease inhibitor that inhibits endogenous tissue-type plasminogen activator (tPA) activation and hence prevents fibrinolysis. PAI-1 is mainly produced by endothelial cells but can also be produced by Adipose tissue. PAI-1 is located in the extracellular matrix and extracellular exosome [45]. Elevated PAI-1 is associated with thrombosis and atherosclerosis [46]. These effects were reversed with the use of Losartan, an AT_1_R blocker. These findings were confirmed in human brain microvascular endothelial cells (HBMECs), in which spike protein increased TF-III and PAI-1 following exposure to hypoxic conditions in HBMECs exposed to hypoxia.

We used a mild chemically induced distal transient MCA thromboembolic model where a clot forms and spontaneously recanalizes within a few hours. However, the model outcomes were significantly changed when animals were pre-injected with SARS-CoV-2 spike protein. Our study showed that pre-injection of SARS-CoV-2 spike protein intensified the decrease in cerebral blood flow and delayed recanalization in the thromboembolic model. These effects may be the result of increased clot formation and reduction in fibrinolysis. Our results showed that SARS-CoV-2 spike protein increased TF-III and PAI expression. Moreover, increased clot formation and delayed recanalization were associated with significant neurological damage, as seen with increased brain infarct size in hACE2 KI mice preinjected with spike protein compared to stroke. Restoration of RAAS balance using AT_1_R blocker, Losartan prevented SARS-CoV-2 spike protein-induced thromboembolic cerebrovascular complications. It is worth mentioning that Losartan treatment was started one week before the induction of stroke. Losartan has been clinically proven to exhibit neuroprotective effects against ischemic stroke [28–30, 47, 48].

Finally, we reported that vascular dysfunction contributes to cognitive impairment and dementia associated with SARS-CoV-2 spike protein-induced thromboembolic stroke. These results agree with Ahmed et al., who showed that the restoration of RAAS via AT_2_R activation contributes to the improvement of cognitive impairments after stroke [49]. We showed that Losartan significantly improved cognitive functions after SARS-CoV-2 spike protein-induced thromboembolic ischemic stroke.

Our study has the following limitations: we have only examined the effect of SARS-COV-2 spike protein on cognitive functions only after short-term induction of stroke. Further investigations are needed to examine the long-term effects of COVID-19. Second, Losartan treatment is started before the induction of stroke, which might have a neuroprotection against ischemic stroke. We only examined the effect of spike protein on endothelial cells. Further studies are required to examine the detrimental effects of spike protein on brain astrocytes, pericytes, and vascular smooth muscle cells.

In conclusion, our study provides new evidence that SARS-CoV-2 spike protein increased coagulation and decreased fibrinolysis in hACE2 KI mice. These effects were accompanied by decreased cerebral blood flow, increased neuronal death, and increased cognitive dysfunctions. Our results showed that restoring RAAS balance using the AT_1_R blocker, Losartan, restored the RAAS balance and reduced COVID-19-induced thromboembolic cerebrovascular complications.

## Data Availability

No datasets were generated or analysed during the current study.
